# Individual Differences in Hemodynamic Responses Measured on the Head Due to a Long-Term Stimulation Involving Colored Light Exposure and a Cognitive Task: A SPA-fNIRS Study

**DOI:** 10.3390/brainsci11010054

**Published:** 2021-01-05

**Authors:** Hamoon Zohdi, Felix Scholkmann, Ursula Wolf

**Affiliations:** 1 Institute of Complementary and Integrative Medicine, University of Bern, 3012 Bern, Switzerland; hamoon.zohdi@ikim.unibe.ch (H.Z.); felix.scholkmann@ikim.unibe.ch (F.S.); 2 Biomedical Optics Research Laboratory, Neonatology Research, Department of Neonatology, University Hospital Zurich, University of Zurich, 8091 Zurich, Switzerland

**Keywords:** systemic physiology augmented functional near-infrared spectroscopy, SPA-fNIRS, colored light exposure, verbal fluency task, cerebral hemodynamics, systemic physiology, laterality

## Abstract

When brain activity is measured by neuroimaging, the canonical hemodynamic response (increase in oxygenated hemoglobin ([O_2_Hb]) and decrease in deoxygenated hemoglobin ([HHb]) is not always seen in every subject. The reason for this intersubject-variability of the responses is still not completely understood. This study is performed with 32 healthy subjects, using the systemic physiology augmented functional near-infrared spectroscopy (SPA-fNIRS) approach. We investigate the intersubject variability of hemodynamic and systemic physiological responses, due to a verbal fluency task (VFT) under colored light exposure (CLE; blue and red). Five and seven different hemodynamic response patterns were detected in the subgroup analysis of the blue and red light exposure, respectively. We also found that arterial oxygen saturation and mean arterial pressure were positively correlated with [O_2_Hb] at the prefrontal cortex during the CLE-VFT independent of the color of light and classification of the subjects. Our study finds that there is substantial intersubject-variability of cerebral hemodynamic responses, which is partially explained by subject-specific systemic physiological changes induced by the CLE-VFT. This means that both subgroup analyses and the additional assessment of systemic physiology are of crucial importance to achieve a comprehensive understanding of the effects of a CLE-VFT on human subjects.

## 1. Introduction

Colored light modulates a wide range of functions in human physiology, including the sleep-wake cycle via melatonin secretion, alertness, cognition, and thermoregulation [1,2]. Since the discovery of the photopigment melanopsin nearly two decades ago, non-image-forming (NIF) vision has been focused on as a potential explanation for a number of effects of colored light on human physiology [3,4]. This light-sensitive protein is expressed in a subclass of intrinsically photosensitive retinal ganglion cells and is most sensitive to narrowband blue light (~460−480 nm) [5,6]. It has become apparent that NIF vision responses to colored light, especially short-wavelength light, affect the cognitive process and enhance alertness and cognitive performance [7,8,9,10,11,12]. However, the effects of colored light on alertness and cognitive performance beyond the scope of NIF vision responses are not yet well understood. Previous studies demonstrated that improvements in cognitive performance via colors or colored light depend not only on the environment and certain situational variables, but also on the individual subject, as well as the type of cognitive task [13,14,15,16].

The verbal fluency task (VFT) is among the most widely applied neuropsychological tests for the assessment of cognitive function. The VFT is a classical method of language production in which subjects are instructed to produce as many words as possible within a restricted time and following specific rules [17,18,19,20,21]. To assess the brain correlates of cognitive functioning underlying the VFT, several studies have been conducted using functional near-infrared spectroscopy (fNIRS) [22,23,24,25,26,27,28,29,30,31]. fNIRS is an optical neuroimaging technique enabling significant advances in the understanding of functional brain activity and higher cognitive functions [32,33]. This non-invasive technique is based on optical spectroscopy and detecting correlates of brain activity mediated by neurovascular coupling (NVC) [34]. A typical NVC response, due to increased neuronal activity, consists of an increase in oxygenated ([O_2_Hb]) and total ([tHb]) hemoglobin and a simultaneous decrease in deoxygenated hemoglobin ([HHb]) [35,36]. fNIRS measurements have demonstrated that the VFT evokes symmetrical cerebral oxygenation responses within different brain regions, primarily in the prefrontal cortex (PFC) and lateral areas [37,38,39,40]. So far, only some studies have reported hemispheric differences in the frontal and temporal cortices, i.e., a more pronounced left compared to right hemispheric activation [41,42,43,44].

Although increases in [O_2_Hb] and decreases in [HHb] serve as indicators for brain activity, other patterns of cortical activation were also found in various cognitive studies [45,46,47]. Such atypical fNIRS patterns can be attributed to different reasons, including individual differences in vascular regulation and measurement positions, as well as influences from changes in systemic physiology [46,48,49,50]. The effects of the latter on changes in [O_2_Hb] and [HHb] can be detected by applying systemic physiology augmented functional near-infrared spectroscopy (SPA-fNIRS), which additionally and concurrently with fNIRS measures systemic physiological parameters [51,52,53].

Since the topic of intersubject variability of hemodynamic responses and changes in systemic physiology is not yet well investigated, we aimed in this study to further explore this topic in detail. In particular, two main goals were pursued: First, we investigated with SPA-fNIRS whether the CLE-VFT causes different reaction patterns in cerebral hemodynamics and systemic physiology. Second, we explored the effects of two light exposure conditions (blue and red) during the CLE-VFT on lateralization of cerebral cortices.

## 2. Materials and Methods

### 2.1. Subjects

The study was conducted with 32 healthy subjects (17 female, 15 male, age 25.5 ± 4.3 years). Subjects were all right-handed with high education level and without any acute or chronic disease affecting the neuronal or cardiorespiratory system. All subjects had normal color vision as assessed by the Ishihara’s Tests for color blindness and color deficiency (Kanehara and CO., LTD., Tokyo, Japan).

### 2.2. Experimental Protocol

**Figure 1:**
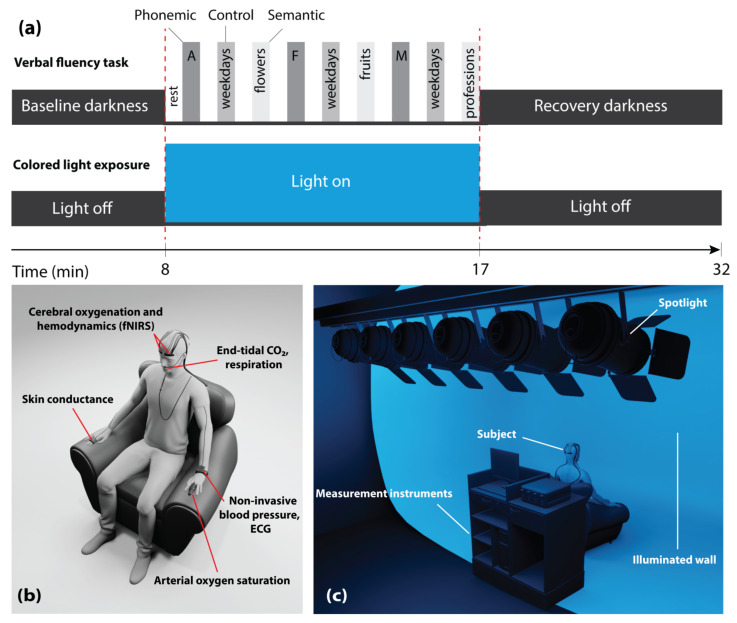
(**a**) Schematic illustration of the measurement protocol; (**b**) visualization of the placement of devices and sensors on the subject; (**c**) experimental setup with the position of the subject, illuminated screen, and spotlights. Two colors, i.e., either red or blue were used for the light exposure; in (**a**,**c**), the case of blue light exposure is visualized.

The subjects sat upright in a comfortable chair in front of a white wall (distance from the subject to the wall: 160 ± 5 cm). Each subject participated in two days of trials: One while being exposed to blue light, and the other while being exposed to red light (illuminance: 120 lux at eye level), the order of which was randomized. On each day, the subjects were exposed to the colored light for a duration of 9 min. The subjects were instructed to keep their eyes open during the entire experiment. Other than during periods of the CLE, the experimental room remained dark. The VFT, which included three sessions, was performed during the CLE. Each session comprised three different trials (phonemic, control, and semantic tasks) in which subjects had to produce as many words as possible for a given letter or category within 30 s. Each trial was followed by a resting phase of 30 s. These periods were set similar to previous VFT protocols in the literature [27,41,44]. Thus, the total duration of the task was 9 min and the period of the CLE was adjusted to this period. Figure 1a presents the schematic representation of the CLE-VFT protocol.

### 2.3. Measurement Setup

The SPA-fNIRS approach includes a multichannel frequency-domain near-infrared spectroscopy (FD-NIRS) system (Imagent, ISS Inc., Champaign, IL, USA) and devices to measure systemic physiological parameters: The SOMNOtouch^TM^ NIBP (SOMNOmedics GmbH, Randersacker, Germany) measured heart rate (HR) with a sampling rate of 4 Hz and mean arterial pressure (MAP) and arterial oxygen saturation (SpO_2_) at a sampling rate of 1 Hz. End-tidal carbon dioxide (P_ET_CO_2_) and respiration rate (RR) were non-invasively measured by NONIN LifeSense (NONIN Medical, Plymouth, MN, USA) at a sampling rate of 1 Hz. An electrodermal activity measurement system (Verim Mind-Reflection GSR, Poland) was employed to determine the skin conductance level (SCL). SCL data were recorded at a sampling rate of 8 Hz. To measure the coupling between HR and RR, the pulse-respiration quotient (PRQ) [54,55] was calculated (PRQ = HR/RR).

The Imagent has 16 laser diodes at 760 nm and 16 laser diodes at 830 nm. Four highly sensitive photo-multiplier tubes are employed as detectors. Each of the four ISS optodes has four light emitters and one light detector, each connected to the instrument by optical fibers. The ISS optodes were placed bilaterally over the prefrontal cortex (PFC) (left: Fp1 and right: Fp2) and the visual cortex (VC) (left: O1 and right: O2) according to the international EEG 10–20 system [56]. The optodes were covered with two layers of dark cloth to prevent ambient light interference. ISS detectors were also shielded against visible light by acrylic long-pass filters with a cut-on wavelength of 685 nm (Knight Optical, Kent, UK) to prevent stray light from affecting the measurement. Moreover, since the light from the ISS instrument is frequency modulated at 110 MHz, exposure light, which is of other frequencies, is removed automatically. The source-detector separations of the optodes were 2.0, 2.5, 3.5, and 4.0 cm over the PFC and 2.0, 2.5, 3.0, and 3.5 cm over the VC. The FD-NIRS system, employing the multi-distance approach, measured absolute [O_2_Hb], [HHb], and tissue oxygen saturation (StO_2_) at a sampling rate of 2.5 Hz on the PFC and VC. The ISS instrument is based on multi-distance frequency domain measurement—which, based on the diffusion approximation, determines the absorption coefficient and the reduced scattering coefficient, and hence, absolute values of [O_2_Hb], [HHb], and StO_2_ [57]. These are calculated online by the software of the instrument.

Figure 1b,c display the positions of the devices and sensors on the subject, as well as the measurement setup showing the position of the subject, the (illuminated) screen, and the spotlights (six LED PAR spotlights—each has 12 × 35 mm RGBW LEDs).

### 2.4. Signal Processing and Statistical Analysis

One subject was excluded from data analysis because she was not a native German/Swiss German speaker. Two subjects aged over 30 years were excluded from the analysis to have a sample in a small age range (20 to 30 years). By removing these two subjects, we avoid the need to correct for an age when performing the statistical analysis, i.e., avoiding to have age-related effects as a confounder. It also helped us to have a quite homogenous sample with respect to the general ability of the subjects (e.g., language proficiency in the VFT task). The language proficiency of the older subjects deviated from the other group. Signal processing was performed in MATLAB (R2017a, MathWorks, Inc., Natick, MA, USA), and statistical analysis in OriginPro (version 2019b, OriginLab Corporation, Northampton, MA, USA)

#### 2.4.1. Signal Processing

Too noisy data were first rejected by manual inspection (e.g., StO_2_ outside the range of 50–100%). At this stage, data with a lower signal-to-noise ratio was mostly found at the VC, due to poor scalp-optode coupling. In total, 80% of the fNIRS signals were accepted for the next data pre-processing step. Then, movement artifacts were removed by the in-house developed movement artifact reduction algorithm based on moving standard deviation and piecewise-interpolation [58]. fNIRS signals were low pass filtered by a robust 2^nd^-degree polynomial moving average (RLOESS) with a window length of 3 min. RLOESS filtering with a window length of > 1 min has been used as a smoothing filter in data processing of the fNIRS signals [51,59,60]. This method was able to remove high-frequency physiological noise (e.g., heart rate and respiratory rate) of the fNIRS signals. The effectiveness of this method has also been shown in the literature [61,62,63]. Furthermore, the FD-NIRS system enabled the measurements to be less sensitive to physiological noise coming from the extracerebral tissue compartment [64]. Then, signals from the left and right PFC and the left and right VC were averaged (since the patterns in the two hemispheres were not significantly different) to obtain signals for the whole PFC and VC, respectively.

All other systemic physiological parameters, except the SCL, were also smoothed using the RLOESS method with a span of 3 min. The SCL data were processed with the Ledalab toolbox [65,66] by means of continuous decomposition analysis performing optimization of 6 initial values.

#### 2.4.2. Data and Statistical Analysis

**Type of functional activation:** The subgroup data analysis was performed by classifying subjects into different groups based on their hemodynamic response pattern of [O_2_Hb] in the PFC and VC during the CLE-VFT. Nine groups are in principle possible, i.e., three directions (increase, no change, decrease in [O_2_Hb]) to the power of two cortices (PFC and VC). To determine the direction during the CLE-VFT phase, the normalized [O_2_Hb] signal was segmented into 40 parts, and the median value for each segment was calculated, followed by applying the one-sample Wilcoxon signed-rank test to all median values of segments. An insignificant (−) pattern indicates a failure to reject the null hypothesis at the 5% significant level, whereas increase and decrease patterns indicate a rejection of the null hypothesis (*p* < 0.05). After classifying each subject into one of the nine groups, all other physiological signals not used for grouping purposes were block-averaged for each group.

**Cerebral functional asymmetry:** The following steps were applied to investigate the functional cerebral oxygenation asymmetry during the CLE-VFT: (i) The time-dependent StO_2_ signal was selected as a promising marker for evaluation of the cerebral laterality. (ii) four different StO_2_ signals from the left and right PFC, and VC were taken into account for each measurement. (iii) each StO_2_ signal was normalized to the last 5 min of the baseline period. (iv) ΔStO_2_ median values during the CLE-VFT were calculated (i.e., Δ indicates the normalized parameter).

**VFT performance:** A total number of VFT correct responses was averaged for all measurements, comprising both the red and blue light exposure. Subjects with a below- and an above-average number of correct responses were allocated to the moderate and excellent performer groups, respectively.

## 3. Results

### 3.1. Subgroup Analysis

While most subjects showed the expected activity pattern in the hemodynamic responses (increase in [O_2_Hb] and decrease in [HHb] at the PFC and VC), a significant number of subjects showed deviations from this pattern. Therefore, the hemodynamic responses were assigned to the nine groups of possible reaction patterns according to the changes of [O_2_Hb] at the PFC and VC (Table 1). In fact, five and seven different hemodynamic response patterns were observed in the subgroup analysis for the blue and red light exposure, respectively.

**Figure 2:**
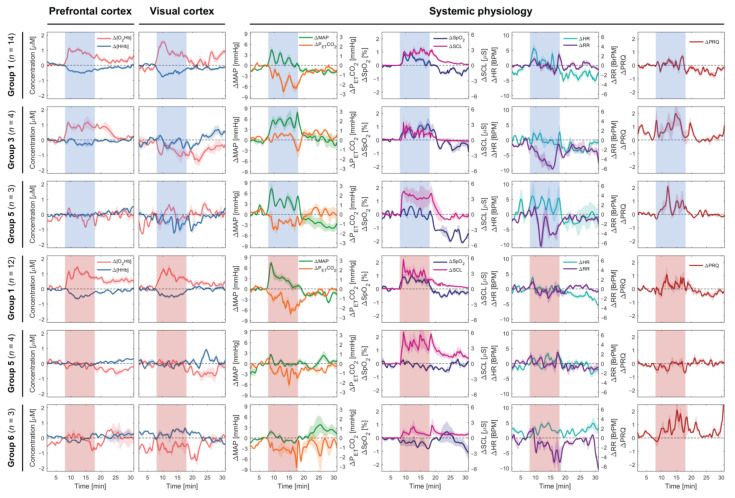
Subgroup analysis (the first three common patterns) of cerebral hemodynamics and systemic physiological parameters evoked by the CLE (blue vs. red light) and VFT. The red and blue shaded areas represent the task/stimulation periods during which the subjects were exposed to the respective colors. Median ± standard error of median (SEM) are shown.

Figure 2 depicts the overview of group-averaged changes in cerebral hemodynamics and systemic physiology based on the first three common [O_2_Hb] patterns at the PFC and VC evoked by the CLE-VFT. Considering the first group (most common pattern) of both colors, the color-dependent changes were only found in HR, which increased during the blue light and was almost constant during the red light exposure.

**Figure 3:**
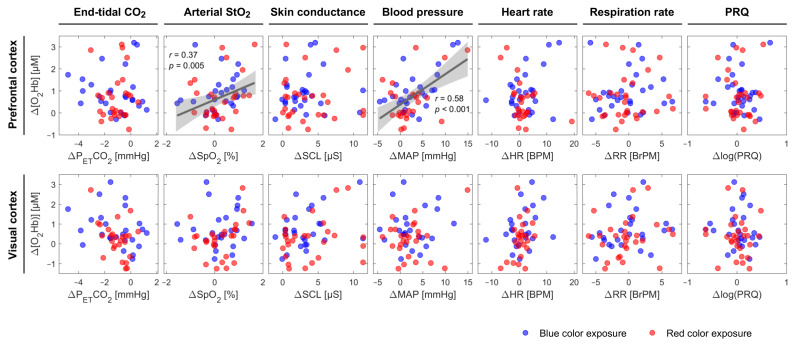
Scatter plots displaying Δ[O_2_Hb] at the PFC and VC vs. other systemic physiological parameters during the CLE-VFT phase at the individual level independent of the color of light. The linear fit is presented for pairs with a significant correlation. The grey shaded areas show 95% of confidence intervals.

An increase in SCL and a decrease in P_ET_CO_2_ were observed for almost all groups independent of the color of light. Apart from fNIRS signals, which were statistically significantly correlated with [O_2_Hb] in the PFC, we found that SpO_2_ and MAP were positively correlated with [O_2_Hb] at the PFC (SpO_2_: *r* = 0.372, *p* = 0.005; MAP: *r* = 0.583, *p* < 0.001) independent of the light’s color and classification of the subjects (Figure 3). In the fifth group of both lighting conditions, color-dependent changes in HR and RR during the CLE-VFT could be observed. HR and RR increased and decreased, respectively, during blue light exposure, while both were constant during red light exposure. Blue light elicited a significant increase in PRQ compared to red light.

### 3.2. Laterality of Cerebral Activity Changes

**Figure 4:**
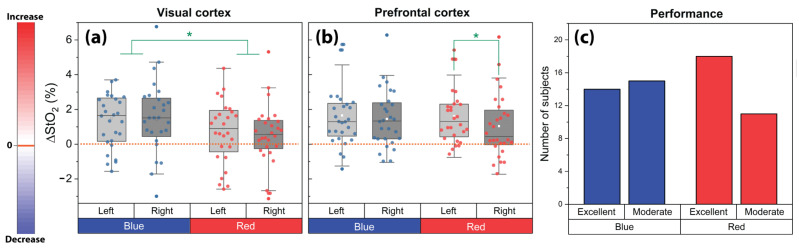
Evoked changes of StO_2_ at the left and right (**a**) VC and (**b**) PFC for the blue and red light exposure during the CLE-VFT; (**c**) Effects of the CLE on the task performance of the subjects. The asterisks indicate the level of significance (*p* < 0.05, Wilcoxon signed-rank test).

Boxplots of ΔStO_2_ values for the left and right VC and PFC and for both conditions (red and blue) during the CLE-VFT were depicted in Figure 4a,b. Evoked changes of StO_2_ were generally higher for the blue light compared to the red light at the VC (*p* < 0.05; effect size (Cohen’s d): *d* = 0.4). Oxygenation response to the CLE-VFT for blue light was bilateral and symmetrical at the PFC, while relatively greater left- than right-hemispheric activation was observed for the red light exposure (*p* < 0.04; *d* = 0.3).

### 3.3. Task Performance

Subjects articulated 56.5 ± 15.1 (mean ± SD; range: 23−100) correct nouns during the blue light exposure and 57.9 ± 11.6 (range: 26−77) during red light exposure. No significant difference in the task performance was found between the blue and red light exposure, and regardless of the color, subjects reached an average number of 57.2 ± 13.4 correct words. This number was taken as a threshold value to classify subjects into two groups (moderate vs. excellent performers). We found that the number of excellent performers during red light exposure was remarkably higher compared to the number of moderate performers, while no difference in the performance of both groups was observed during blue light (Figure 4c). Moreover, there was a significant difference between excellent performers during blue light versus red light conditions. In other words, the difference between the sample standard deviation of excellent performer groups under the influence of blue and red light is big enough to be statistically significant (F-Test: 3.72, *p* = 0.015).

## 4. Discussion

### 4.1. Prefrontal Cerebral Oxygenation Asymmetry during the Red Light Exposure

The lateralization of brain function is a propensity for some neural functions or cognitive processes specialized to one side of the cortex or the other. Numerous studies have provided valuable insights into the cerebral asymmetry of the human brain cortices [67,68,69]. In particular, the frontal lobe has increasingly become a special region of interest. Frontal cerebral asymmetry of resting-state brain activity has been explained using the approach-withdrawal model, where the higher relative leftward frontal activity is associated with appetitive motivation and approach-related affect (positive affect), while the rightward frontal activity is related to behavioral inhibition and withdrawal-related affect (negative affect) [51,70,71,72]. In the present study, we show that during the CLE-VFT, red light caused higher oxygenation in the left PFC compared to the right. The left relative to right frontal cortical activation during the red light might be attributed to greater positive affect, according to the approach-withdrawal model. Interestingly, we also found that the number of excellent performers during the red light exposure was remarkably higher than the number of moderate performers. In other words, red light led to better performance of subjects in the VFT, which showed its impact on leftward prefrontal lateralization. Our finding (that there is a positive correlation between the number of excellent performers and relative leftward PFC activity) is in accordance with previous research [42] which concluded that during the VFT, subjects with excellent task performance showed a left-dominated dorsolateral frontal asymmetry, while moderate performers showed a right-dominated frontopolar asymmetry [42]. Moreover, slightly better VFT performance with red as compared to blue light exposure is in line with many color and colored light studies which have proposed that red improves task performance in comparison with other colors [73,74,75,76,77]. In particular, red enhances the performance for “overlearned motor”, “proofreading”, “target shooting”, and “basic strength” tasks [76,78,79,80]. It has been demonstrated that red facilitates performance on detail-oriented tasks that require concentration and careful attention, while blue improves performance on creative tasks [80,81]. It was also presented that a low-demand task worsens performance in blue rather than red environments [75]. Beyond task type, other factors, including the subject’s emotional state, subject’s personality, subject’s color preferences, and the subject’s culture, may also influence cognitive performance. For example, better performances in color conditions lead to higher arousal [82]. In another study, the impact of color depended on personality [83]. For example, high screeners, i.e., people who have a natural tendency to effectively reduce the complexity of an environment, performed better in a red-painted office, whereas low screeners benefited from blue-green office spaces [83].

### 4.2. Other Patterns As the Typical Hemodynamic Response Pattern were Observed in Half of the Subjects

We selected [O_2_Hb] as a marker to classify subjects into different groups. Compared to [HHb], this parameter is a more sensitive marker of cerebral blood flow (CBF) changes [49,84]. Besides, it has an acceptable high reproducibility, as well as a higher signal-to-noise ratio in comparison with the [HHb] signal [49,85,86].

Cognitive activation normally leads to an increase in [O_2_Hb] and a decrease in [HHb], which is known as a typical hemodynamic response pattern. Despite this typical pattern normally observed at the group level, our subgroup analysis showed that this pattern was found in approximately 50% of cases (blue: 14 out of 25 cases, 56%; red: 12 out of 26 cases, 46%). The remaining subjects showed different cortical activation patterns. In total, five and seven different hemodynamic response patterns were detected in the subgroup analysis of the blue and red light exposure, respectively. We already reported in another study that the blue light exposure, without any cognitive test, led to 8 different hemodynamic response patterns (*n* = 32, age: 23.8 ± 2.2, 15 min blue light exposure at 120 lux illuminance) [47]. A possible explanation for the lower number of classified groups in this experiment (compared to the previous research [47]) could be that in this study, it is very likely that the attention of the subjects was mainly focused on the VFT and the stimulating impact of CLE decreases when the brain is already involved in a challenging VFT condition. Therefore, the more prominent impact of VFT compared to CLE caused less variety of hemodynamic response patterns.

We also found that an increase and a decrease in [O_2_Hb] at the PFC and VC, respectively, was the second most common pattern during blue light, which is interestingly in line with our previous study [47]. Sakatani et al. also observed three different patterns of fNIRS parameter changes during a mental stress task [87]. They found that the frequency of the typical cortical activation response in a younger group (*n* = 24, age: 21.3 ± 0.9, 80% of subjects) was noticeably higher than in an older group (*n* = 11, age: 56.9 ± 4.2, 55% of subjects). Moreover, Quaresima and Ferrari reported that the typical hemodynamic response to the VFT was observed in only 4 out of 8 cases [45]. Consequently, based on the results of this research and the above-mentioned studies, it is clear that in spite of the typical cortical activation response (normally observed at the group-level), not all subjects react the same, and atypical changes in fNIRS signals can also be detected. One possible explanation for intersubject-variability of the responses is the fact that the environment and certain situational variables may influence cortical activation response. The dependence of cerebral parameters on several factors, including seasonal changes, time of day, temperature, mood, and chronotype, was investigated in detail in our recent paper [51]. Briefly, we showed that absolute values of StO_2_ during the resting state were not correlated with season and subjects’ mood, but with the time of day and subjects chronotype [51]. Furthermore, we observed that frontal cerebral oxygenation asymmetry was correlated with the season and room temperature, but not dependent on subjects chronotype [51]. For this study, it was tried to keep all the experimental conditions constant. For example, two factors, including the date and time of participation, were precisely controlled for each subject’s two participations. In terms of lighting conditions, all subjects experienced the same situation at least one h before the measurement. All measurements were also carried out in a time period from late August to early September, and the room temperature was almost constant (range: 22.4 °C to 22.9 °C). Therefore, in the current study, it seems that situational variables had minimal effects on the intersubject-variability of cerebral hemodynamic responses. However, atypical cortical activation responses can be triggered by diverse neuroanatomy, partial volume effects, variations in CBF, and systemic physiology [47,48,49]. For an in-depth explanation of the reasons for atypical pattern occurrence, the readers are kindly directed to Holper et al. [46].

### 4.3. SpO_2_ and MAP are Positively Correlated with [O_2_Hb] at the PFC

Different physiological sources may cause false-positives and false-negatives in fNIRS signals [88]. The recorded physiological signals, thus, can be used to regress out the components of systemic physiological signals from the brain signals measured by fNIRS. These include changes in blood pressure parameters, P_ET_CO_2_, SpO_2_, and activity of the sympathetic nervous system [50]. It is also known that the systemic parameters are interrelated with the metabolic changes in the brain [51,52,53], and atypical changes in fNIRS signals can be triggered by systemic physiological factors [50,88,89]. Therefore, it is essential to employ the SPA-fNIRS approach to ensure the correct interpretation of changes in cerebral hemodynamics and oxygenation.

Considering the first group (i.e., the most common pattern) for both light colors (blue and red), we found color-dependent changes in HR, i.e., an increase during blue and no change during red. Although not all subjects showed an increase in HR during blue light, the increase found in most cases may be associated with the autonomous nervous system responding to light with an increase in sympathetic tone (short-wavelength light) [90]. Besides, independent of the color type and classification of the subjects, a decrease in P_ET_CO_2_ and an increase in SCL were observed for all groups, which once again shows that the effects of VFT on these two physiological signals were more dominant than the CLE effects. The decline in P_ET_CO_2_ during the CLE-VFT is in line with the research that the effect of different speech tasks on P_ET_CO_2_ was studied [91]. The lower CO_2_ pressure is most likely attributed to the changes in breathing (hyperventilation) during the VFT. The increase in SCL can also be caused by various factors, namely, stress, which can be triggered by challenging VFT.

We found that SpO_2_ was positively correlated with [O_2_Hb] at the PFC independent of color type and classification of the subjects. Although several studies demonstrated a significant positive correlation between SpO_2_ and cerebral (or somatic) tissue oxygen saturation [92,93,94,95,96], it is to the best of our knowledge that this is the first study showing a positive correlation between SpO_2_ and [O_2_Hb] at the PFC during a functional paradigm. [O_2_Hb], measured by fNIRS, mainly reflects O_2_Hb in small arteries, capillaries, and veins in brain tissue [97]. Lindauer et al. stated that variations in SpO_2_, as well as other factors, including changes in CBF, intracranial pressure, and systemic pressure, may be the reasons for atypical cortical activation responses [48]. The MAP may also be accounted as a biomarker describing the [O_2_Hb] changes at the PFC (positively correlated with [O_2_Hb] at the PFC). This should be interpreted with care, since there is one exception for group 5 of the blue light (Figure 2), where [O_2_Hb] was constant and MAP increased significantly. This can be attributed to the small number of subjects allocated to this group of blue light exposure. A large number of subjects may have revealed a correlation between MAP and [O_2_Hb] in this group. MAP is an important parameter used to avoid false-positive results and to identify real cerebral hemodynamics and oxygenation changes [88]. A correlation between MAP and [O_2_Hb] (or StO_2_) has been reported in various functional studies [89,98,99]. Tsubaki et al. investigated the relationships between NIRS signals and MAP during exercises on a bicycle ergometer [100]. They found highly significant correlations between MAP and [O_2_Hb] during warm-up and at workloads corresponding to 30 and 50% of peak oxygen consumption [100]. In another recent study, in contrast, a non-significant association between StO_2_ and MAP was observed in critically ill adults [101].

Considering the fifth group of both conditions, there were color-dependent changes in HR and RR during the CLE-VFT. HR and RR increased and decreased, respectively, during the blue light, while both were constant during the red light. It has been reported elsewhere that colored light had no effects on RR [102,103]. Because of the large intersubject variation caused by subjects having different RR, this parameter should always be interpreted with caution. We also found that the blue light exposure evoked a significant increase in the PRQ compared to red light in the fifth group of both conditions. This can be explained mostly by an increase in HR (or decrease in RR). The PRQ is a useful and unitless parameter to attain the overall state of human physiology [54]. No statistically significant changes in the PRQ in response to the CLE were observed at the group level of the study conducted by Edelhäuser et al. [102]. However, in a study of short-term CLE conducted by our group, blue light exposure caused a decrease in the PRQ [53].

In general, there are three possible explanations for the observed correlation between fNIRS signals, namely, [O_2_Hb], and systemic physiology, such as SpO_2_ and MAP. (i) The fNIRS signals of the brain are caused by changes in systemic physiology. (ii) The systemic physiological changes are caused by brain activity. (iii) The fNIRS signals reflect indeed NVC only, and the correlation we found between [O_2_Hb] and systemic physiology has no causal relation.

One possibility for the first explanation is the fact that low-frequency changes (e.g., Mayer waves and task-evoked changes, due to systemic physiological activity) were not removed by the filtering, and hence, they are visible in both the systemic and cerebral variables. Although it is often assumed that fNIRS purely detects the cerebral-evoked-neural response in the brain, in reality, each fNIRS signal contains different components [88]. Still, in our opinion, the appropriate explanation relies probably on a mixture of the three above-mentioned effects, i.e., there is a complex interrelation of systemic physiology and brain activity. In our data, this is visible by—on the one hand—a slightly greater change in the VC compared to the PFC, indicating that fNIRS detects brain activity. On the other hand, the correlations between fNIRS and systemic physiological signals indicate that the fNRIS signals are also influenced by systemic changes.

### 4.4. How does [O_2_Hb] Behave During Continuous Long-Term Stimulation?

Further investigating long-term colored light exposure is a crucial strategy needed to study and understand human physiology, especially in our modern society, when we are extensively exposed to different colored light. So far, few studies have investigated how brain activity changes during continuous long-term colored light stimulation [104,105]. It was shown that there is a habituation effect in the brain’s activity, and this *habituation* is reflected as decreased oxygenation during the visual stimulation [106,107]. On the other hand, oxygenation may remain elevated (*plateau*) during long visual stimulation, decreasing only when the flow rate decreases, attributing to neuronal habituation effects [108]. One study using fNIRS also indicated that during continuous visual stimulation, [O_2_Hb] increased during the first 19 s of stimulation and reached a *plateau*, and remained constantly elevated during the entire 5 min of the activation period [109]. In our study, both above-mentioned effects are visible, but they apply to different groups of subjects. For example, in the first group, during the blue CLE-VFT, the [O_2_Hb] at both cortices decreased, therefore, indicating *habituation*. In the third group, during the same condition, [O_2_Hb] reached a *plateau* during the CLE-VFT and even remained elevated at the beginning of the recovery phase. Similar trends are also visible during red CLE in group 1. The MAP shows similar trends during blue CLE, i.e., *habituation* in group1 and a *plateau* in group 3. Interestingly, P_ET_CO_2_ shows a decrease (away from baseline) in group 1 during blue CLE, while it remains mostly unchanged in group 3. This may indicate that the *habituation* seen in group 1 is merely due to a CO_2_ response. Thus, our study found changes in systemic physiology that impact the fNIRS signals and strongly enhance the understanding of changes in cerebral hemodynamics and oxygenation. Moreover, it is difficult to provide a concrete interpretation of previous fNIRS studies carried out without measurements of systemic physiology. Therefore, it is our opinion that measurements of systemic physiology along with cerebral hemodynamics are essential and should be carefully considered when performing neuroimaging studies.

## 5. Conclusions

We found that red light exposure led to better performance of subjects taking the VFT, while simultaneously showing a physiological response of higher oxygenation in the left PFC than the right.

Furthermore, we demonstrated that stimulus-evoked changes in cerebral hemodynamics, oxygenation, and systemic physiological activity generally show large intersubject variability. This means that each subject displayed individual responses to the experimental paradigm. A group-level analysis, although commonly used, only reveals the most prominent tendency between subjects: It is unable to account for the individual variability and consequently impedes a comprehensive and correct conclusion. Therefore, the subgroup or subject-specific analysis is needed to completely understand the effects of a CLE-VFT. Despite the typical hemodynamic response pattern (increase in [O_2_Hb] and decrease in [HHb]) normally observed at the group level, the subgroup analysis showed that this pattern was found in only ~50% of the cases and the number of these typical hemodynamic response patterns was different between the red and blue light exposure. Our systemic physiology augmented fNIRS (SPA-fNIRS) approach enabled us to determine that SpO_2_ and MAP correlate with the changes in [O_2_Hb] at the PFC during the CLE-VFT, i.e., that systemic and cerebral physiology interact. This shows the importance of assessing systemic physiology in addition to neuroimaging to enable a comprehensive understanding of changes in cerebral hemodynamics and oxygenation. It also demonstrates that individuals respond differently to colored light not only on the cerebral, but also on the systemic level. This individual variability needs to be taken into account, in particular, when considering the influence of colored light on daily human life, e.g., at the workplace or in public places.

## Figures and Tables

**Table 1:** Classification of the hemodynamic response of [O_2_Hb] patterns (significant increase ↑, insignificant change –, significant decrease ↓) at the PFC and VC.

	Cerebral Cortex		Number of Subjects	
	PFC	VC	Blue Light Exposure	Red Light Exposure
**Group 1**	↑	↑	14 (7 female, 7 male)	12 (4 female, 8 male)
**Group 2**	↑	–	2 (1 female, 1 male)	2 (1 female, 1 male)
**Group 3**	↑	↓	4 (3 female, 1 male)	1 (1 female, 0 male)
**Group 4**	–	↑	2 (0 female, 2 male)	2 (2 female, 0 male)
**Group 5**	–	–	3 (2 female, 1 male)	4 (2 female, 2 male)
**Group 6**	–	↓	-	3 (2 female, 1 male)
**Group 7**	↓	↑	-	2 (1 female, 1 male)
**Group 8**	↓	–	-	-
**Group 9**	↓	↓	-	-

## Data Availability

The data that support the findings of this study are available from the corresponding author upon reasonable request.
